# Supplementary material for: Association of Chrononutrition Indices with Anthropometric Parameters, Academic Performance, and Psychoemotional State of Adolescents: A Cross-Sectional Study
Source: Nutrients. 2023 Oct 25;15(21):4521. doi: 10.3390/nu15214521 (PMC10647400; doi:10.3390/nu15214521)
Supplement: Supplementary file 1 [file nutrients-15-04521-s001.zip › nutrients-2658789-supplementary.pdf]

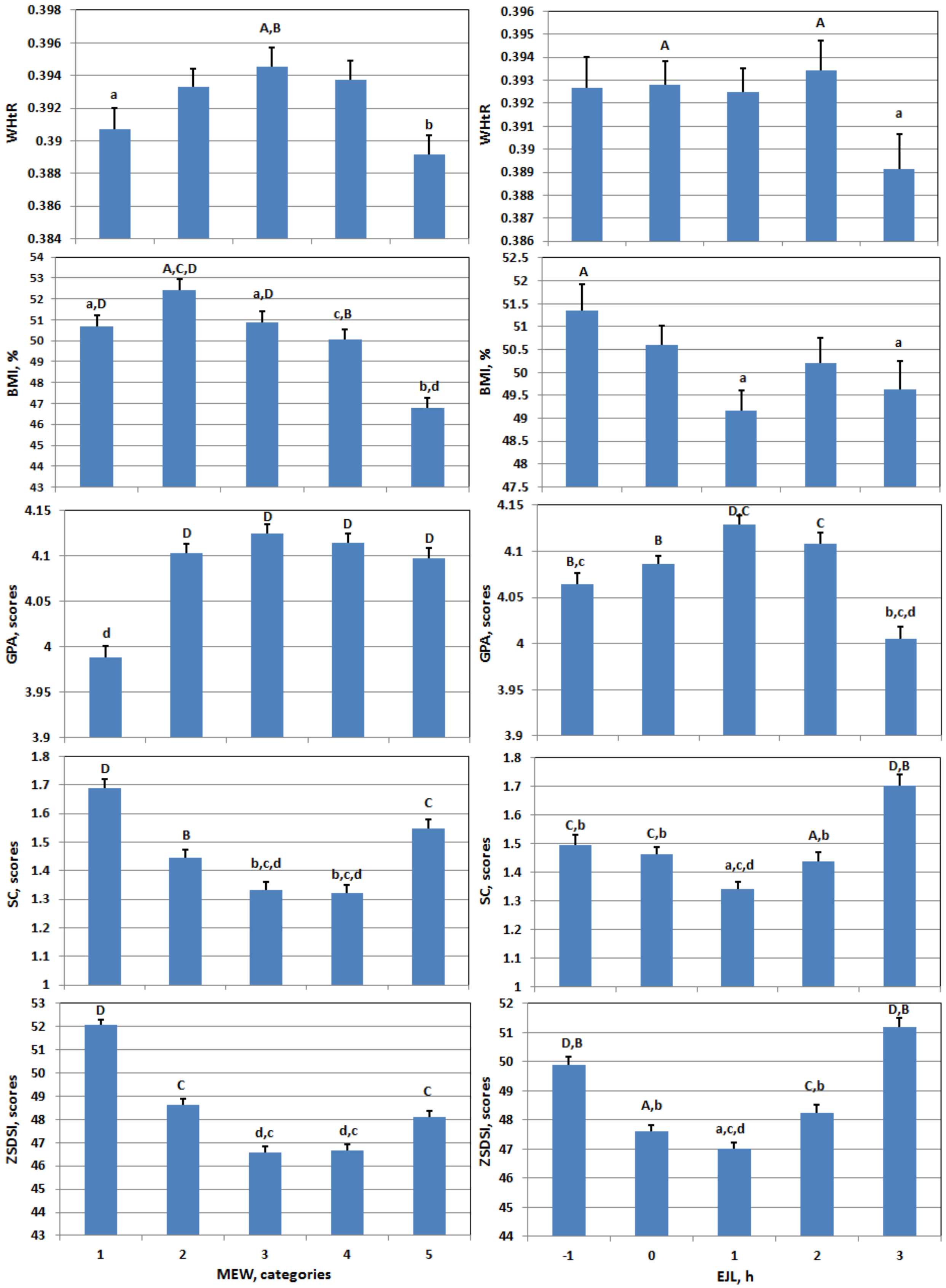

Figure S1. The association of meal timing characteristics with the anthropometric, cognitive, and psychoemotional state of schoolchildren.

MEW: mean weekly eating window; EJL: eating jetlag; EPFc: eating mid-phase on free days corrected for week/free days variability; NE: night eating; WHtR: waist-to-height ratio; BMI, %: body mass index, percentiles; GPA: academic performance; SC: symptom counts of food addiction; ZSDSI: depression. Differences between group indicated by letters are significant (Student test: A>a, $p < 0.05$; B>b, $p < 0.01$; C>c, $p < 0.001$; D>d, F>f, G>g, H>h, $p < 0.0001$; E>e, $p < 0.00001$)

Figure S1 Continue

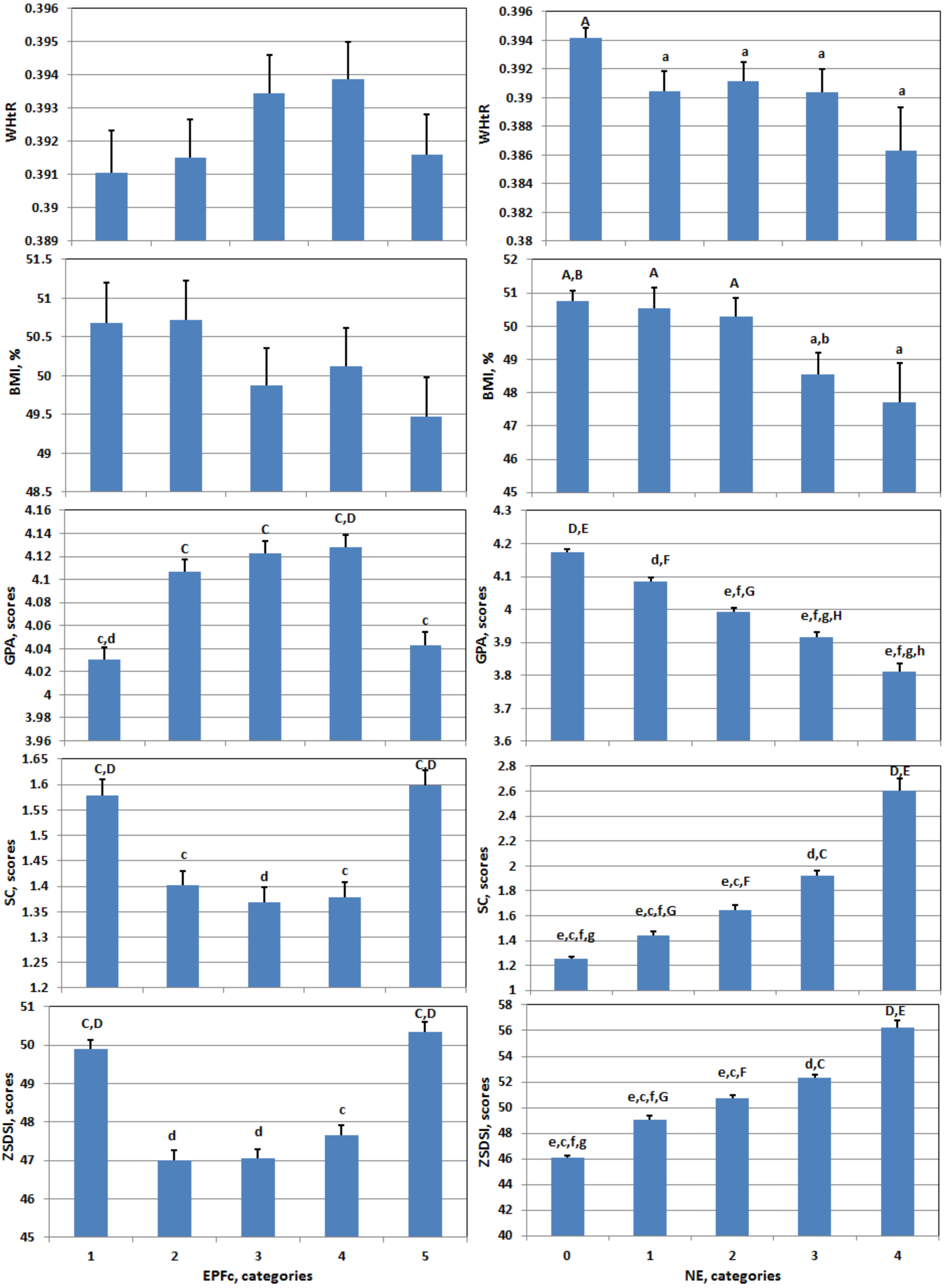

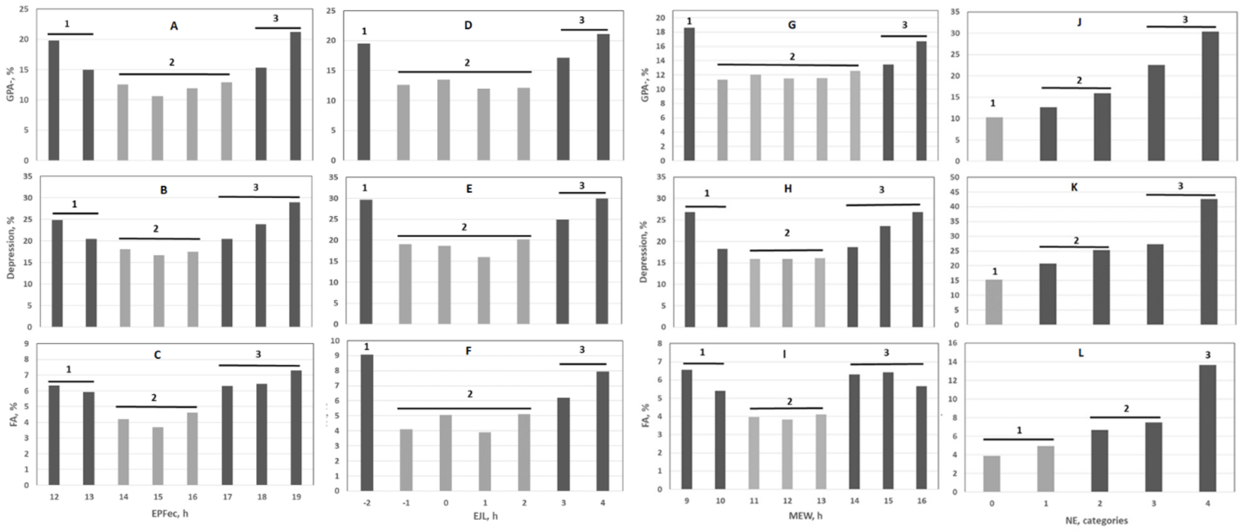

Figure S2. The association of meal timing characteristics with the categorical anthropometric, cognitive, and psychoemotional state of schoolchildren. The minimum values of indicators of academic performance (percentage of students with "satisfactory" academic performance, GPA_L, %), depression (%) and food addiction (FA, %) are highlighted in light.

Table S1. The association of meal timing characteristics with the categorical anthropometric, cognitive, and psychoemotional state of schoolchildren.

					**1/2**			**3/2**		
		**1**	**2**	**3**	χ^2	***P***	φ	χ^2	***P***	φ
EPFec	GPA-	17.79	11.89	18.02	59.13	0.000	0.075	44.86	0.000	0.069
	Depression	23.05	17.31	23.47	37.29	0.000	0.065	48.03	0.000	0.072
	FA	6.17	4.14	6.59	15.60	0.000	0.042	24.85	0.000	0.052
EJL	GPA-	19.47	12.65	18.93	26.74	0.000	0.051	45.72	0.000	0.064
	Depression	29.60	18.17	27.17	54.28	0.000	0.073	67.88	0.000	0.079
	FA	9.05	4.59	6.98	26.68	0.000	0.052	15.51	0.000	0.038
MEW	GPA-	18.61	11.75	14.56	94.43	0.000	0.091	4.34	0.050	0.023
	Depression	24.57	15.99	21.07	114.44	0.000	0.107	21.85	0.000	0.056
	FA	6.25	3.96	6.25	26.93	0.000	0.052	14.35	0.000	0.045
		**1**	**2**	**3**		**2/1**			**3/1**	
NE	GPA-	10.30	14.45	24.58	37.34	0.000	0.061	262.42	0.000	0.175
	Depression	15.26	23.21	31.17	94.74	0.000	0.098	241.03	0.000	0.170
	FA	4.07	7.02	13.65	42.21	0.000	0.061	93.77	0.000	0.106

The figures indicate groups of indicators expressed as a percentage, as in Figure S2.

A chi squared-test was used for analysis of the association of meal timing characteristics with categorical variables (Female, %, BMI categories, %, ZSDSI categories, %, and FA, %). Cramer's V (φ) was used as a measure of effect size for categorical variables.
